# The status quo of short video as sources of health information on gastroesophageal reflux disease in China: a cross-sectional study

**DOI:** 10.3389/fpubh.2024.1400749

**Published:** 2024-05-28

**Authors:** Ying Cai, Hao Zeng, Pingping Yang, Xiwen Xu, Yongkang Lai, Yu Zhou

**Affiliations:** ^1^Department of Gastroenterology, Gaoxin Branch of The First Affiliated Hospital of Nanchang University, Nanchang, Jiangxi, China; ^2^Department of Gastroenterology, Shanghai Changhai Hospital, Naval Medical University, Shanghai, China

**Keywords:** gastroesophageal reflux disease, health information, TikTok, Bilibili, short videos

## Abstract

**Background:**

Positive lifestyle adjustments have become effective methods in treating gastroesophageal reflux disease (GERD). Utilizing short video platforms to encourage GERD patients for effective self-disease management is a convenient and cost-effective approach. However, the quality of GERD-related videos on short video platforms is yet to be determined, and these videos may contain misinformation that patients cannot recognize. This study aims to assess the information quality of GERD-related short videos on TikTok and Bilibili in China.

**Methods:**

Search and filter the top 100 GERD-related videos on TikTok and Bilibili based on comprehensive rankings. Two independent gastroenterologists conducted a comprehensive evaluation of the video quality using the Global Quality Score and the modified DISCERN tool. Simultaneously, the content of the videos was analyzed across six aspects: definition, symptoms, risk factors, diagnosis, treatment, and outcomes.

**Results:**

A total of 164 GERD-related videos were collected in this study, and videos from non-gastrointestinal health professionals constitute the majority (56.71%), with only 28.66% originating from gastroenterology health professionals. The overall quality and reliability of the videos were relatively low, with DISCERN and GQS scores of 2 (IQR: 2–3) and 3 (IQR: 2–3), respectively. Relatively speaking, videos from gastrointestinal health professionals exhibit the highest reliability and quality, with DISCERN scores of 3 (IQR: 3–4) and GQS scores of 3 (IQR: 3–4), respectively.

**Conclusion:**

Overall, the information content and quality of GERD-related videos still need improvement. In the future, health professionals are required to provide high-quality videos to facilitate effective self-disease management for GERD patients.

## Introduction

Gastroesophageal reflux disease (GERD) is an inflammatory condition of the esophagus caused by gastroesophageal reflux, which can be diagnosed across all age groups, with a global prevalence estimated to be between 8 and 33% (1). Symptoms of GERD may include gastric burning or regurgitation, non-cardiac chest pain, and numerous extra-esophageal symptoms such as cough, voice difficulties, and throat pain (2). In recent years, the prevalence of symptomatic GERD has been gradually increasing, significantly impacting patients’ quality of life, escalating economic burdens, and raising the risk of esophageal stricture and adenocarcinoma (3, 4). Therefore, addressing the treatment and management of GERD has become a crucial issue in global healthcare.

Most non-severe cases of GERD can typically be improved by optimizing lifestyle, proton pump inhibitors (PPIs) treatment, and appropriate adjunctive medication (2). However, studies have reported that up to 50% of patients do not achieve sufficient relief from empirical PPI therapy, and long-term PPI use not only imposes a significant economic burden on patients but also leads to a series of complications (1, 5). Therefore, proactive lifestyle changes and weight management have gradually become key and cost-effective approaches to the treatment of GERD. Eherer et al. (6) found that actively training the diaphragm through respiratory exercises significantly improved gastroesophageal reflux, thereby reducing the disease burden of reflux gastroesophageal reflux. Wang et al. (7) through a retrospective analysis of the lifestyles of 689 patients, discovered that habits such as overeating, a preference for sweets, and sleeping with a low pillow height increase the occurrence of gastroesophageal reflux disease. Singh et al. (8) conducted an intervention on the weight of 332 GERD patients, and the results showed that weight loss could reduce the gastric reflux symptom scores in over 80% of the participants. Unfortunately, many doctors either do not provide specific guidance on lifestyle changes or give patients a printed list of activities and foods, making it challenging for patients (1, 9). Thus, providing accurate and effective guidance for lifestyle changes is crucial for reducing the incidence of GERD and improving the prognosis of GERD patients.

With the continuous development of technology, emerging internet technologies such as short video platforms are improving communication methods between doctors and patients, providing patients with more convenient answers and health education (10–12). Social networking platforms like TikTok and Bilibili disseminate information in the form of vivid and easily understandable graphic videos. Ordinary individuals can quickly access desired health information by searching keywords on these platforms, gradually replacing traditional text-based information such as newspapers or books (13, 14). Additionally, health information in visual formats can evoke emotional responses in consumers and motivate healthy actions (15). Therefore, health education for patients through short video platforms is expected to be a major trend in future public health information dissemination. However, despite the enormous potential of short videos, the complexity of original literature and the heterogeneity of internet information sources result in significant variations in the quality of health-related information on short video platforms (13, 14, 16). Many low-quality videos contain inaccurate or biased information that may mislead patient judgment and even have negative impacts on health, especially for lifestyle-influenced diseases like GERD. To the best of our knowledge, the quality of GERD-related information on video-based social media has not been thoroughly assessed. Thus, this study aims to investigate the information quality of GERD videos on TikTok and Bilibili in China.

## Methods

### Search strategy and data collection

This is an observational retrospective study conducted from January 10, 2024, to January 15, 2024, retrieving the top 100 videos on two of China’s most popular short video platforms, TikTok and Bilibili. The search keywords were “胃食管反流病” (“Gastroesophageal reflux disease” in Chinese) or “反流性食管炎” (“reflux esophagitis” in Chinese). The exclusion criteria were as follows: (1) Non-Chinese videos; (2) Duplicate content videos; (3) Videos without sound or poor sound quality; (4) Videos unrelated to the topic; and (5) Videos used for advertising or commercial purposes. Two qualified doctors (YC and XX), working in the gastroenterology department of tertiary hospitals, independently reviewed and categorized the videos. They have extensive experience in managing patients with gastroesophageal reflux disease. In case of any discrepancies, they resolved them through consensus and consultation.

Excel (Microsoft Corporation) was utilized to gather information on each video, including the publication date, uploader’s name and type, medical department, video duration (in seconds), and the counts of likes, favorites, and shares.

### Evaluation methodologies and procedure

This study assessed the content and information quality of the videos using the modified DISCERN tool and the Global Quality Score (GQS). DISCERN, a widely validated and applied tool, was employed to assist consumers and healthcare professionals in evaluating the reliability of health-related content in videos (13, 17). Originally proposed by Goobie et al., the tool primarily evaluates videos through five questions (refer to Supplementary Table 1). Scores for these questions are assigned “1” or “0” based on whether the answer is “yes” or “no,” with a minimum score of 0 out of 5. The GQS (16, 18), another extensively used tool for evaluating the quality of health information in videos, adopts a five-point scale based on the quality of health information in the video, ranging from 1 (poor quality) to 5 (good quality) (Detailed information about this tool is available in Supplementary Table 2). Additionally, we scored six main aspects of video content: disease definition, signs and symptoms, risk factors, assessment, management, and outcomes. Scores were categorized into five main sections: no content (0 points), minimal content (0.5 points), some content (1 point), substantial content (1.5 points), and extensive content (2 points).

Before video screening, all settings and history records on the smartphone were deleted to avoid potential directed video recommendations caused by the pre-buffering cache. During the screening process, no videos were downloaded, forwarded, liked, or commented on to prevent data interference. Two researchers independently completed the video screening and rating process. Initially, the study recorded video information, including the publication date, duration, number of likes, favorites, and shares, as well as publisher information, such as account name, self-description, and verification status. Based on the uploader’s account name and verification status, they were categorized into two main types (health professionals and non-health professionals). Health professionals were further divided into gastroenterology professionals (certified physicians or practicing nurses) and non-gastroenterology professionals. Non-health professionals were categorized into News agencies/Nonprofit organizations and Science Blogger. Finally, two independent raters evaluated the content, reliability, and quality of the videos using the DISCERN and GQS scoring systems. Before the assessment, both raters reviewed the official descriptions of the tools mentioned above, discussed the optimal way to use these tools to assess video content, and made any necessary adjustments.

### Statistical analysis

Categorical variables were expressed as frequencies and percentages, and differences were analyzed using the chi-square test or Fisher’s exact test. For normally distributed data, continuous variables were presented as mean ± standard deviation, and analysis was conducted using Student’s *t*-test. For non-normally distributed data, continuous variables were represented as the median and interquartile range (IQR) for skewed data, and the Mann–Whitney U test was employed for analysis. All statistical analyses were conducted using R statistical software version 4.2.3.^1^

## Results

### Video characteristics

**Figure 1:**
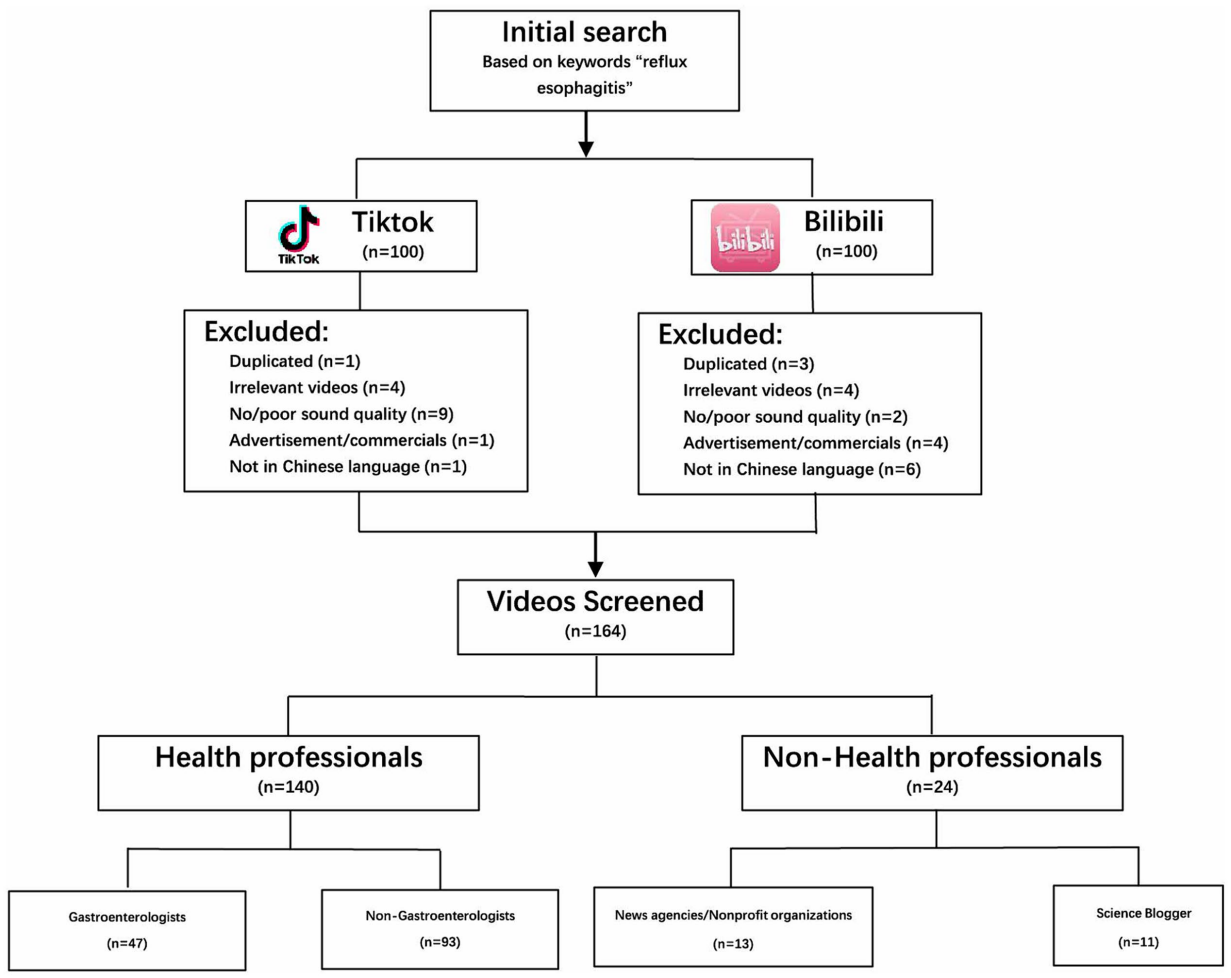
The flow chart of GERD-related videos included in the present study.

Based on the inclusion and exclusion criteria, a total of 164 videos were screened from the two platforms (Figure 1). According to the primary uploaders of the videos, they were categorized into health professionals and non-health professionals. Videos published by health professionals accounted for 85.37% (140/164), while those by non-health professionals constituted 14.63% (24/164). Further analysis of health professionals revealed that videos from non-gastroenterology sources predominated, accounting for 56.71% (93/164), with only 28.66% (47/164) originating from health professionals in the field of gastroenterology. Non-health professionals mainly derived from News agencies/Nonprofit organizations and science bloggers, accounting for 7.92% (13/164) and 6.71% (11/164) of the total videos, respectively (Table 1).

**Table 1:** Characteristics of reflux esophagitis–related videos videos.

Characteristics	*n* = 164
Source	
Health professionals	
Gastroenterologists, No. (%)	47 (28.66)
Non-gastroenterologists, No. (%)	93 (56.71)
Non-Health professionals	
News agencies/Nonprofit organizations, No. (%)	13 (7.92)
Science Blogger, No. (%)	11 (6.71)
Video duration (seconds), range	16–1,351
Video duration (seconds), median, IQR	88.5 (62.5–178.5)
Number of likes, median, IQR	284 (36–3041.5)
Number of shares, median, IQR	75.5 (13–733)
Number of favorites, median, IQR	112 (21–878)
DISCERN score, median, IQR	2 (2–3)
GQS score, median, IQR	3 (2–3)

The duration of the videos ranged from 16 to 1,351 s, with a median duration of 88.5 s (IQR: 62.5–178.5). During the study period, the median number of likes received by the collected videos was 284 (IQR: 36–3041.5). The median numbers of favorites and shares for the videos were 75.5 (IQR: 13–733) and 112 (IQR: 21–878), respectively. Further comparative analysis of videos from sources such as gastroenterology, non-gastroenterology, News agencies/Nonprofit organizations, and science bloggers revealed significant differences. Videos from science bloggers had a significantly higher median duration of video (median: 254, IQR: 129–341) compared to videos from other sources, while videos from gastroenterologists received significantly higher likes (median: 608, IQR: 98–8,449), shares (median: 144, IQR: 31–3,454), and favorites (median: 172, IQR: 48–2027.5) than videos from other sources (Table 2). Comparative analysis of videos sourced from TikTok and Bilibili, as shown in Table 3, demonstrated that the median duration of videos (median: 131, IQR: 79–271) from Bilibili was significantly greater than those from TikTok. However, the number of likes (median: 36, IQR: 8–172), favorites (median: 27, IQR: 7–91), and shares (median: 17, IQR: 3–46) was significantly lower for videos from Bilibili compared to those from TikTok.

**Table 2:** Comparison of reflux esophagitis-related video characteristics according to video source.

Video features	Gastroenterologists	Non-gastroenterologists	News agencies/Nonprofit organizations	Science Blogger	*p*
Video duration (seconds), median, IQR	79 (61–136)	82 (61–143)	179 (105–341)	254 (129–341)	0.003
Number of likes, median, IQR	608 (98–8,449)	353 (36–3,153)	17 (2–204)	179 (46–445)	0.001
Number of shares, median, IQR	144 (31–3,454)	88 (11–683)	6 (0–70)	65 (18.5–264)	0.007
Number of favorites, median, IQR	172 (48–2027.5)	123 (16–1,118)	14 (1–69)	113 (20.5–309.5)	0.004
DISCERN score, median, IQR	3 (3–4)	2 (2–3)	2 (2–2)	2 (2–2.5)	<0.001
GQS score, median, IQR	3 (3–4)	3 (2–3)	3 (2–3)	2 (1.5–3)	<0.001

**Table 3:** Comparison of reflux esophagitis-related video characteristics on two platforms.

Video features	TikTok (*n* = 83)	Bilibili (*n* = 81)	*p*
Video duration (seconds), median, IQR	70 (52–109.5)	131 (79–271)	<0.001
Number of likes, median, IQR	2,846 (582.5–9619.5)	36 (8–172)	<0.001
Number of favorites, median, IQR	608 (137–3,136)	27 (7–91)	<0.001
Number of shares, median, IQR	619 (135.5–3177.5)	17 (3–46)	<0.001
DISCERN score, median, IQR	2 (2–3)	2 (2–3)	0.885
GQS score, median, IQR	3 (2–3)	3 (2–3)	0.393

### Video content analysis

This study evaluated video content from six aspects: definition, signs and symptoms, risk factors, diagnostics, treatment, and outcomes. As shown in Table 4, a few videos provided a comprehensive overview of GERD. Overall, videos tended to focus more on describing the symptoms of GERD, with 37.2% containing extensive symptom-related content, following risk factors (28.7%), definition (26.8%), and treatment (26.8%). 53% of the videos did not mention the diagnostic methods for GERD, and 47.6% did not address the outcomes of GERD.

**Table 4:** Completeness of video content.

Video content	Definition, *n* (%)	Signs/symptoms, *n* (%)	Risk factors, *n* (%)	Test, *n* (%)	Treatment/Management, *n* (%)	Outcomes, *n* (%)
No content (0 points)	43 (26.2)	12 (7.3)	51 (31)	87 (53)	27 (16.5)	78 (47.6)
Little content (0.5 points)	28 (17.1)	22 (13.4)	18 (11)	13 (7.9)	15 (9.1)	27 (16.5)
Some content (1 point)	25 (15.2)	49 (29.9)	30 (18.3)	39 (23.8)	50 (30.5)	24 (14.6)
Most content (1.5 points)	24 (14.6)	20 (12.2)	18 (11)	13 (7.9)	28 (17.1)	16 (9.6)
Extensive content (2 points)	44 (26.8)	61 (37.2)	47 (28.7)	12 (7.4)	44 (26.8)	19 (11.7)

**Figure 2:**
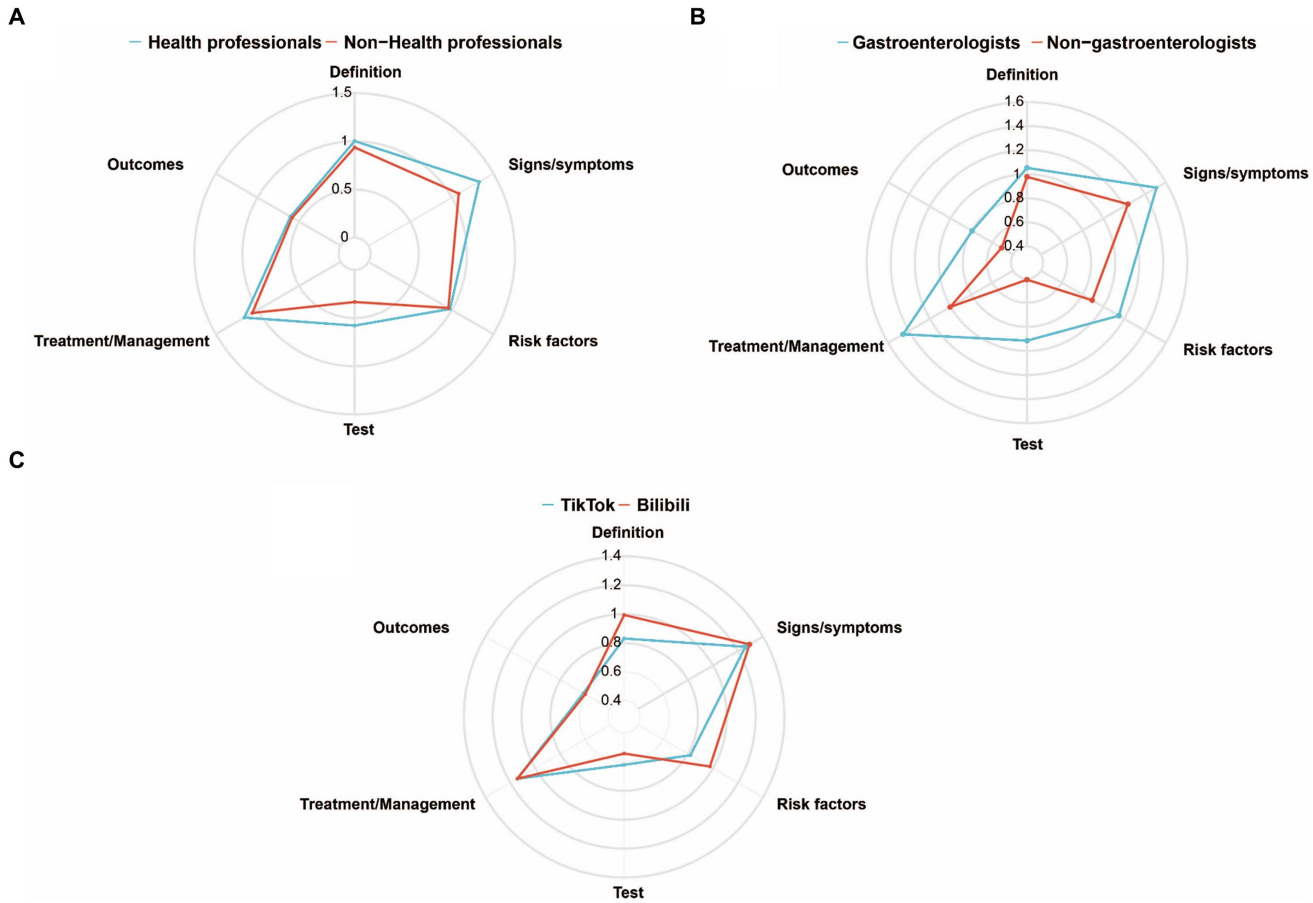
Comparison of content comprehensiveness between health professionals and non-health professionals **(A)**; gastroenterologists and non-gastroenterologists **(B)**; and Tiktok and Bilibil **(C)**.

Subgroup analysis based on the video uploader was conducted. As illustrated in Figure 2A, videos from health professionals, in general, contained more content in terms of definition, signs and symptoms, risk factors, diagnostics, treatment, and outcomes compared to those from non-health professionals. Within health professionals, videos from gastroenterology included more content in all aspects compared to those from non-gastroenterology. However, both gastroenterology and non-gastroenterology source videos provided relatively less content regarding outcomes. Among them, the gastroenterology source videos had more content on treatment (Figure 2B). Comparing videos from TikTok and Bilibili sources, the results indicated that Bilibili-sourced videos covered more content on the definition, symptoms, and risk factors of GERD than those sourced from TikTok, while TikTok-sourced videos had slightly more content on the test than Bilibili-sourced videos (Figure 2C).

### Video quality and reliability assessment

**Figure 3:**
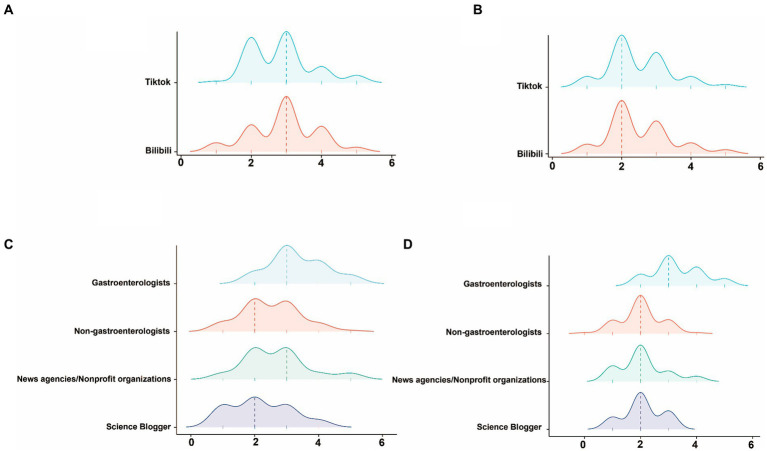
The distribution of DISCERN scores **(A)** and GQSs **(B)** between Tiktok and Bilibil displayed by the ridge plot. The distribution of DISCERN scores **(C)** and GQSs **(D)** among gastroenterologists, non-gastroenterologists, news agencies/nonprofit organizations, and science bloggers displayed by the ridge plot.

In summary, the overall quality and reliability of the videos were relatively low, with DISCERN and GQS scores of 2 (IQR: 2–3) and 3 (IQR: 2–3), respectively(Table 1). Further subgroup analysis revealed that videos from gastroenterology had the highest reliability, with DISCERN scores of 3 (IQR: 3–4), significantly surpassing those from non-gastroenterology, News agencies/Nonprofit organizations, and Science Bloggers (*p* < 0.001). In terms of overall quality assessment, videos from gastroenterologists were also significantly higher than videos from other sources, with GQS scores of 3 (IQR: 3–4). Videos published by Science Bloggers exhibited the lowest quality and reliability in this study (Table 2; Figure 3). Additionally, a comparison was made between the quality and reliability of videos from the two major platforms. The results indicated no significant difference in the quality and reliability of videos sourced from TikTok and Bilibili (Table 3; Figure 3).

## Discussion

Overall, the reliability and quality of GERD-related videos on these two platforms in China are not high. There are hardly any videos that comprehensively cover all six aspects of GERD, including definition, symptoms, risk factors, testing, treatment, and outcomes. This limited coverage may reduce the utility for patients seeking information on GERD.

Gastroesophageal reflux disease is one of the most common chronic diseases globally, associated with a reduced health-related quality of life and increased risk of severe complications (2). Since the late 1990s, the prevalence of GERD has been on the rise due to increased gastric acid secretion, decreased *Helicobacter pylori* infection rates, and westernized lifestyles (4, 5, 19). The clinical management of GERD significantly impacts the lives of many patients and has substantial implications for healthcare and society. Empirically, PPIs are a practical initial diagnostic approach, suitable for patients with typical GERD symptoms and those with atypical symptoms (chronic cough, hoarseness, non-cardiac chest pain, and wheezing) (2). However, the widespread and inappropriate use of PPIs is a prevalent issue, leading to suboptimal outcomes in GERD treatment. Lifestyle modifications represent another crucial therapeutic avenue for GERD and are a non-pharmacological and self-manageable approach for effective treatment (1). However, patients need a certain level of awareness and understanding of GERD before implementing these changes. For instance, weight management and adjustments in diet/nighttime habits can increase intra-abdominal to intra-thoracic pressure gradients, reducing the occurrence of reflux (20, 21); elevating the head of the bed and avoiding meals within 3 h before bedtime can decrease reflux, especially in patients known to have hiatal hernia or symptomatic burdens during postprandial or nocturnal periods (22). However, due to the strained medical resources in China, many GERD patients cannot obtain detailed disease health education or care plans from clinical professionals. This situation is exacerbated, especially during the COVID-19 pandemic (23, 24). Therefore, accessing disease-related information via the Internet has become a crucial avenue for health education in the current landscape.

Short video platforms are currently a popular form of internet tool (13, 14, 16). During the coronavirus pandemic, the viewership of videos related to the pandemic on the short video platform TikTok reached 931 billion by July 2020 (25). Studies indicate that health-related short videos on TikTok have garnered over 1.7 million likes and 176,000 comments, suggesting that short videos have gradually become a primary means for patients to acquire health-related information (26). However, the quality of videos varies, and some may even disseminate incorrect health information, which could lead patients to exert more effort in treating GERD, or improper guidance on PPI usage may worsen the patient’s condition. Previously, Aydin et al. and Naga Nyshita et al. evaluated the quality of GERD-related videos on YouTube, and their results consistently indicated the poor quality of GERD content on this platform (27, 28). However, there is currently no research assessing the quality of related short videos on Chinese platforms such as TikTok and Bilibili. Therefore, it is crucial for healthcare professionals to review the content of videos in China and screen for high-quality information, ensuring its effectiveness in patient health education and disease self-management.

Short videos have become an important source for educating patients about disease management, making it imperative to establish rules for posting health education short videos on relevant online platforms to safeguard public health. In this study, we compared the content and quality of short videos from different sources and found that although videos from health professionals constitute the majority, the overall quality of the videos remains suboptimal. This is primarily attributed to the contribution of videos from non-gastroenterology, accounting for nearly half of the videos. Non-gastroenterologists still possess some inaccuracies in their understanding of and insights into GERD, affecting the overall quality. While videos from gastroenterology exhibit higher quality and reliability compared to videos from other sources and are more popular, they predominantly provide accurate information on how patients can self-manage GERD through diet and lifestyle habits. However, there is a scarcity of videos informing patients about how to undergo testing for GERD and the prognosis of GERD patients. Additionally, we observed that some videos from popular science bloggers exaggerated occasional adverse reactions to GERD treatment medications, potentially misleading the public. Our findings can help patients seeking GERD information on platforms like TikTok and Bilibili find higher-quality videos. Moreover, it can guide patients to adopt correct lifestyle changes, which can reduce healthcare costs and alleviate the disease burden on society.

Compared to traditional text information, short videos provide patients with disease-related information in a graphical video format, making it easier for users to absorb and remember complex health information (17). However, incorrect health information in short videos can make self-management of diseases extremely challenging. Therefore, improving the information quality of short videos is crucial for maintaining patient health and reducing the economic burden on patients. Based on the results of this study, we propose the following suggestions for the future use of short videos as a tool for health education: Firstly, relevant authorities should increase supervision and management of health education content in short video releases. Professionals should carefully review the content before users publish health education-related information through short videos. Secondly, relevant authorities should organize and advocate for experienced experts in the field to release authoritative videos on relevant diseases. Certified authoritative videos could even be prioritized as the first video shown to patients after a search, reducing the likelihood of patients being misled by other incorrect information. Finally, relevant government bodies, professional organizations, and experts should actively counteract misinformation. The general public must selectively consume credible videos to enhance their judgment and seek guidance from reliable sources. In addition, recent research has confirmed that machine learning plays an important role in identifying video quality (29, 30). Therefore, in the future, developing a plugin that can only recognize the quality of videos uploaded by users through machine learning methods seems to be an effective way to achieve video quality supervision.

This study has the following limitations. Firstly, the evaluation of video quality in this study was limited to the two most commonly used short video platforms in China, and the quality of videos on other platforms remains to be further investigated. Secondly, this study focused on Chinese video content, and videos from other language sources, such as YouTube, were not included. Therefore, the results of this study may not be applied to videos in other languages. Finally, due to privacy considerations, accurate view counts for the videos in this study were challenging to obtain. However, based on the number of likes, shares, and bookmarks, we can still conclude that these videos have a considerable viewership on Chinese short video platforms, and therefore, the results of this study remain reasonably accurate.

## Conclusion

Overall, the quality of GERD-related short videos is not entirely satisfactory, and short video platforms still have a high number of low-quality videos. The quality of the videos was related to the source of the videos, with gastroenterologists posting videos with higher DISCERN scores and GQS scores compared to other sources. In terms of content, few videos provide comprehensive details about GERD, and while videos by gastroenterologists can accurately inform patients about how lifestyle changes can improve and treat GERD symptoms, it is noteworthy that there is a lack of videos detailing specific tests to assess GERD disease status and describing what the prognosis of GERD is. In the future, there is a need to organize the publication of more authoritative short videos related to GERD, as well as to strengthen the management and supervision of related videos to more accurately disseminate related knowledge to patients, thereby enhancing self-management of GERD patients.

## Data availability statement

The original contributions presented in the study are included in the article/Supplementary material, further inquiries can be directed to the corresponding author.

## Author contributions

YC: Conceptualization, Data curation, Formal analysis, Funding acquisition, Investigation, Methodology, Project administration, Resources, Supervision, Visualization, Writing – original draft, Writing – review & editing. HZ: Writing – original draft, Writing – review & editing. PY: Data curation, Writing – review & editing. XX: Data curation, Writing – original draft. YL: Formal analysis, Writing – original draft. YZ: Writing – original draft.

## Footnotes

^1^ www.r-project.org
