# Supplementary material for: The status quo of short video as sources of health information on gastroesophageal reflux disease in China: a cross-sectional study
Source: Front Public Health. 2024 May 28;12:1400749. doi: 10.3389/fpubh.2024.1400749 (PMC11165113; doi:10.3389/fpubh.2024.1400749)
Supplement: Supplementary file 1 [file Table_1.docx]

**Supplementary Table 1: Modified DISCERN quality criteria for assessing the reliability of video. (1 point for answer ‘yes’, 0 point for answer ‘no’)**

| **Reliability Score** |
| --- |
| 1. Is the video clear, concise, and easy to understandable? |
| 2. Are valid sources referenced? |
| 3. Is the presented content balanced and impartial? |
| 4. Are other content sources listed for patient reference? |
| 5. Was uncertainty addressed in any areas? |
