# Supplementary material for: The status quo of short video as sources of health information on gastroesophageal reflux disease in China: a cross-sectional study
Source: Front Public Health. 2024 May 28;12:1400749. doi: 10.3389/fpubh.2024.1400749 (PMC11165113; doi:10.3389/fpubh.2024.1400749)
Supplement: Supplementary file 2 [file Table_2.docx]

**Supplementary Table 2: Global Quality Score (GQS) (Scoring ranges from 1 to 5)**

| **GQS Definition** | **Score** |
| --- | --- |
| Poor quality, video streaming is choppy, most information is missing, and it's of no use to patients | 1 |
| Generally poor quality and poor flow, the quality is poor, the flow is not smooth, some information is listed, but many important topics are omitted, making it very limited for patients' use | 2 |
| Moderate quality, with sufficient discussion on some important information | 3 |
| Good quality good flow, covering most relevant information, useful for patients | 4 |
| Excellent quality and flow, very useful for patients | 5 |
